# Adaptive Contrastive Learning with Label Consistency for Source Data Free Unsupervised Domain Adaptation

**DOI:** 10.3390/s22114238

**Published:** 2022-06-02

**Authors:** Xuejun Zhao, Rafal Stanislawski, Paolo Gardoni, Maciej Sulowicz, Adam Glowacz, Grzegorz Krolczyk, Zhixiong Li

**Affiliations:** 1 CRRC Academy Co., Ltd., Beijing 100070, China; xuejunzhao0@gmail.com; 2 Department of Electrical, Control and Computer Engineering, Opole University of Technology, 45-758 Opole, Poland; r.stanislawski@po.opole.pl; 3 Department of Civil and Environmental Engineering, University of Illinois at Urbana-Champaign, Champaign, IL 61820, USA; gardoni@illinois.edu; 4 Department of Electrical Engineering, Cracow University of Technology, 31-155 Cracow, Poland; maciej.sulowicz@pk.edu.pl (M.S.); adglow@agh.edu.pl (A.G.); 5 Faculty of Mechanical Engineering, Opole University of Technology, 45-758 Opole, Poland; g.krolczyk@po.opole.pl; 6 Yonsei Frontier Lab, Yonsei University, Seoul 03722, Korea

**Keywords:** unsupervised domain adaptation, contrastive learning, source free domain adaptation

## Abstract

Unsupervised domain adaptation, which aims to alleviate the domain shift between source domain and target domain, has attracted extensive research interest; however, this is unlikely in practical application scenarios, which may be due to privacy issues and intellectual rights. In this paper, we discuss a more challenging and practical source-free unsupervised domain adaptation, which needs to adapt the source domain model to the target domain without the aid of source domain data. We propose label consistent contrastive learning (LCCL), an adaptive contrastive learning framework for source-free unsupervised domain adaptation, which encourages target domain samples to learn class-level discriminative features. Considering that the data in the source domain are unavailable, we introduce the memory bank to store the samples with the same pseudo label output and the samples obtained by clustering, and the trusted historical samples are involved in contrastive learning. In addition, we demonstrate that LCCL is a general framework that can be applied to unsupervised domain adaptation. Extensive experiments on digit recognition and image classification benchmark datasets demonstrate the effectiveness of the proposed method.

## 1. Introduction

Deep neural network [1,2,3,4] has achieved remarkable success in different application scenarios, but the excellent performance of deep learning comes from large-scale data annotation and long-time model training. In order to avoid expensive labeling cost and training time, domain adaptation is proposed to make full use of previously labeled data sets and unlabeled target domain datasets, and has achieved competitive results in the fields of image recognition, object detection semantic segmentation and so on.

In the last decade, many scholars have conducted extensive research on domain adaptation, especially in the scene of unsupervised domain adaptation [5,6]. The most classic strategy in unsupervised domain adaptation is to align the domain distribution. These works achieve domain alignment between the source domain and the target domain through various metrics, such as maximum mean dispersion [7] and Wasserstein dispersion [8]. Another popular framework [5] is based on a domain adversarial network, which aims to learn domain invariant features to minimize the discrepancy between the two domains.

In recent years, due to privacy issues and intellectual rights, the training data cannot be directly accessed. These existing unsupervised domain adaptation often requires source data, which may violate the policy of data privacy protection. In this paper, we discuss a practical and challenging source-free unsupervised domain adaptation, which uses the model trained in the source domain to adapt to the target domain. Specifically, only the model trained by the source domain and the unlabeled data of the target domain is provided. Our goal is to obtain knowledge from the source domain model and target domain data, so as to adapt the model to the target domain and obtain competitive performance.

In source-free unsupervised domain adaptation, most methods are influenced by SHOT [9] and use pseudo labeling technology for self-training. We believe that a good classification model should meet two key conditions: (1) the class weight is located in the class feature center in the feature space; (2) category semantic information should be discriminative. In fact, the pseudo-label-based method only meets the first one, without considering the second one. We believe that learning the distinguishability semantic representation of unlabeled data can promote network adaptation together with pseudo labels. Contrastive learning affirms that the samples of the same class should be closer and the samples of different classes should be farther. In standard contrastive learning, two related views of the same image can be naturally compared. Recently, some works have introduced contrastive learning into domain adaptation and achieved good results. In source-free unsupervised domain adaptation, due to data privacy and other reasons, we cannot obtain the data of the source domain, so we cannot directly apply contrastive learning between the source domain and the target domain; however, if we obtain the feature of the same version of the credible historical version of the sample, we can make a better contrast; therefore, domain adaptation may benefit from our contrastive learning.

In view of this, we introduce LCCL, a simple but effective contrastive learning framework in source-free unsupervised domain adaptive scenarios. Given the source domain model and target domain data, due to the discrepancy between domains, we use information maximization to alleviate domain differences. Owing to the lack of labels in the target domain, we use pseudo labeling technology to give pseudo labels, so as to promote the self-training process. In order to make full use of trusted pseudo labels, we select the features of samples with consistent network prediction and pseudo labels to store in the memory bank. We minimize the distance between samples and samples of the same class in the memory bank and maximize the distance between samples of different classes. Through this mechanism, we can fully explore the structural information of the target domain and better adapt the source domain model to the target domain.

In brief, we highlight our three-fold contributions.

- An adaptive contrastive learning framework that works at the class level for source-free unsupervised domain adaptation is proposed.
- The proposed method introduces a memory bank that stores reliable samples with consistent labels and encourages samples in the target domain to learn discriminative features at the class level.
- Comprehensive experiments show that our method is competitive with existing methods in a series of source-free unsupervised domain adaptation scenarios.

## 2. Related Work

Unsupervised domain adaptation (UDA) has been widely studied in recent years. Most of the existing methods [5,10,11,12,13,14,15] solve the domain adaptation problem by reducing domain discrepancy or adding adaptation layers to match feature distributions. For example, DDC [12] uses moment matching to align feature distributions. DANN [5] and MCD [16] learn domain invariants by designing domain discriminators. Not long ago, someone introduced the prototype method and contrastive learning to solve the UDA problem. For example, TPN [17] tries to align the source domain and target domain through the learned prototype feature representation. In addition, CAN [18] and CoSCA [19] methods use contrastive learning to reduce the inter domain intra-class distance and maximize the inter-class distance; however, due to privacy issues, the source data may not be available in practice, so these methods cannot be used in the source free scenario.

Source-free unsupervised domain adaptation (SFUDA) aims to adapt the network to the target domain without the source domain data. There are two main methods at present, one is the pseudo labeling method represented by SHOT [9], and the other is the method of generating target style image represented by MA [20]; however, directly using the pseudo labels in the target domain is very easy to produce the problem of noise amplification. On the other hand, it is very difficult to directly generate the target style image in the source model. Recently, BAIT [21] introduced additional classifiers to find the features of misclassification. When the feature extractor is updated, these features will be pushed to the right of the source decision boundary, so as to realize source free unsupervised domain adaptation.

Contrastive learning (CL) is a self-supervised learning method, which helps the model learn the discriminative feature between samples. Generally speaking, it is to make the distance between similar samples smaller and the distance between different samples larger in the feature space. Recently, various works [22,23] have shown that the selection of data is very important for contrastive learning. There are generally two strategies in unsupervised learning. One is to use clustering to pseudo label unlabeled data [24], so as to guide the pair reconstruction. The other is to start from multiple perspectives, using the augmentation of samples to construct data pairs [25]. The augmentations of the same sample are its positive pairs and other samples are negative pairs. After a given data pair, some contrastive learning losses are also proposed. Triple loss [26] is widely used in face recognition, minimizing the distance between the anchor and positive, and maximizing the distance between the anchor and negative. NCE [27] regards the problem as a binary classification problem. The classifier can binary classify data samples and noise samples, and this classifier is what we need. Contrastive learning does not need to pay attention to the details at the pixel level, but only needs to learn to distinguish the data in the feature space at the semantic level; therefore, the model and its optimization become simpler and have a stronger generalization ability.

**Figure 1:**
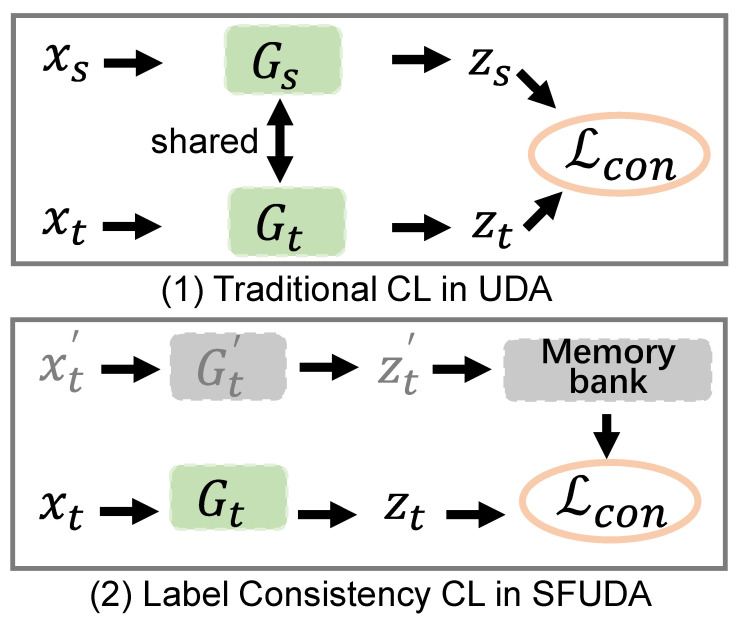
Our label consistency contrastive learning is different from the traditional contrastive learning in unsupervised domain adaptation.

Comparison with existing work. For the classical SFUDA, it is obvious that we differ from the existing work, because we propose a new framework to introduce contrastive learning into the scene of source-free methods. Compared with the existing contrastive learning [28] in the traditional unsupervised domain adaptation, we draw the differences in Figure 1. Unsupervised domain adaptation generally uses all the data of source domain and target domain to participate in contrastive learning. When we cannot access the source domain data, we add the reliable samples with consistent labels into the memory bank as the keys, which can reduce the impact of noise pseudo labels on the performance of domain adaptation.

## 3. Methodologies

**Figure 2:**
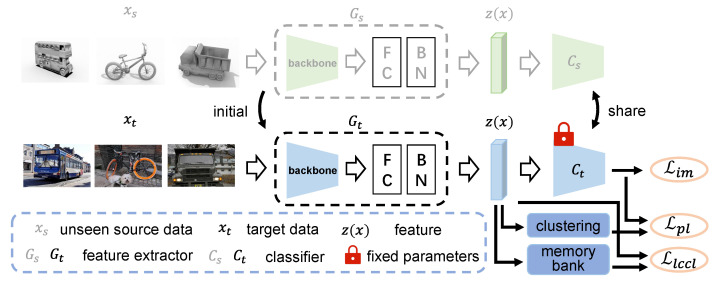
The pipeline of our LCCL framework. The source model consists of the feature learning module and the classifier module. LCCL fix the weight parameters of classifier and utilizes the feature learning module as initialization for target domain learning. The method includes three losses, information maximization loss $L_{im}$, pseudo labeling loss $L_{pl}$ and label consistency contrastive learning loss $L_{lccl}$.

**Problem definition**. In unsupervised domain adaptation, there are two domains with different distributions: source domain and target domain. Here, we consider a *K*-class classification task in which the source domain and the target domain share the same label space. In source-free unsupervised domain adaptation, the data in the source domain are invisible, and only the model trained in the source domain can be provided. Our goal is to train a network, which can be divided into feature extractor *G* and classifier *C*. For a sample *x*, the feature after passing through the feature extractor is z(x)=G(x), and the final output of the network is p(x)=δ(C(G(x))), where δ is the softmax function. The pipeline of our LCCL framework is shown in Figure 2.

**Information maximization loss**. In UDA, many classical methods try to align different domains through matching data distribution, which use maximum mean discrepancy [7] or domain adversarial network [5]. In SFUDA, we also hope to learn a better target feature extractor to align the feature distribution of source domain and target domain; however, we have no access to the source domain data. On the other hand, if the distribution discrepancy between the source domain and the target domain is alleviated, the output of unlabeled data in the target domain should be similar to one-hot encoding; therefore, we introduce information maximization loss, which can make the output of individual samples in the target domain more confident, and make the whole have diversity to reduce the problem of long tail. The formula of information maximization loss is as follows, including entropy loss and diversity loss.

(1) $$\begin{array}{c} L_{im}=-E_{x\in X_{0}}\sum_{k=1}^{K}p\left(x\right)\log p\left(x\right)\\ +\sum_{k=1}^{K}{\bar{p}}_{k}\log{\bar{p}}_{k}. \end{array}$$

where $\bar{p}\;=\;E_{x\in X_{0}}\left[p\left(x\right)\right]$ is the mean of the softmax outputs for the current batch.

**Pseudo labeling loss**. Although maximizing the loss of information can achieve a more reliable prediction of the target domain, it will inevitably be affected by wrong pseudo tag matching. In order to solve this problem, a general method is to use pseudo tag technology for self-training and select more accurate pseudo labels to further promote the migration effect of the network. In fact, we learn from the idea of k-means. Specifically, we first calculate the centroid of each class by weighted k-means.

(2) $$\mu_{k}^{\left(0\right)}=\frac{\sum_{x\in X}p\left(x\right)z\left(x\right)}{\sum_{x\in X}p\left(x\right)},$$

where $\mu_{k}^{\left(0\right)}$ is the initial center for k-means, p(x) is the soft labels, z(x) is the feature generate from the encoder. The centroid obtained for the target domain can better represent the distribution of the target domain, resulting in more robust results.

Then we can give the sample pseudo label through the centroid of the nearest neighbor.

(3) $${\hat{y}}_{t}=\arg\min_{k}D\left(z\left(x\right),\mu_{k}^{\left(0\right)}\right),$$

where D(a,b) measures the cosine distance between *a* and *b*.

The process of obtaining the centroid by clustering and re-assigning the pseudo label will last for multiple rounds. Finally, our pseudo label can be obtained through the final class centroid.

(4) $$\begin{array}{c} \mu_{k}^{\left(1\right)}=\frac{\sum_{x\in X}\xi \left({\hat{y}}_{t}=k\right)z\left(x\right)}{\sum_{x\in X}\xi \left({\hat{y}}_{t}\right)},\;\;\;\\ {\hat{y}}_{t}=\arg\min_{k}D\left(z\left(x\right),\mu_{k}^{\left(1\right)}\right). \end{array}$$

where ξ(∗) is an indicator that produces 1 when the argument is true, ${\hat{y}}_{t}$ are the final calculated pseudo labels. As we all know, the cyclic calculation of K-means to re-assign pseudo labels is carried out in multiple rounds, which is set as two round in our experiment.

Given the pseudo label, the loss function can be calculated by the standard cross-entropy loss.

(5) $$L_{pl}=-E_{x_{t}\in X_{t}}\sum_{k=1}^{K}\xi \left({\hat{y}}_{t}=k\right)\log p\left(x\right).$$

**Label consistency contrastive learning loss**. Due to the lack of source domain data and target domain labels, the proposed label consistency contrastive learning learns the distinguishability relationship with historical model samples from unlabeled target samples. The loss we use is the standard infoNCE loss, and the formula is defined as:

(6) $$L_{lccl}\;=-\log\frac{\exp(\phi \left(q,k^{+}\right)/\tau )}{\exp\left(\phi \left(q,k^{+}\right)/\tau \right)+\sum_{j=1}^{K-1}\exp\left(\phi \left(q,k_{j}^{-}\right)/\tau \right)}.$$

where q denotes a sample in the target domain. The key value is the historical characteristics of the samples stored in the memory bank. $k^{+}$ is the sample set of the same class as the query samples in the memory bank, and $k^{-}$ is the sample set of all classes different from the query samples in the memory bank. It should be noted that the size of the memory bank is fixed to L. When updating, it is the same as the queue storage. The latest sample features are sent to the queue, and the features at the end of the queue are eliminated. Moreover, ϕ(a,b) denotes the cosine similarity and τ is a temperature factor.

In order to obtain a more reliable key set, so as to improve the performance of contrastive learning, under the influence of DTFLC [29], in each minibatch, we select samples of the consistency between the labels given by clustering and the labels given by the network. The formula is as follows.

(7) $${\hat{y}}_{t}=\arg\max_{k}p{\left(x\right)}_{k}.$$

Only when the conditions of the formula are met, we add these samples to the memory bank to learn better feature representation.

Overall, the total loss function can be formulated as follows:

(8) $$L=L_{im}+\alpha L_{pl}+\beta L_{lccl}.$$

In order to better understand our algorithm, we also list the flow of our algorithm in Algorithm 1.
**Algorithm 1** LCCL algorithm for SFUDA task.**Input:** source model $f_{s}=G_{s}\circ C_{s}$, target data $x_{i=1}^{n_{t}}$, maximum number of epochs $T_{m}$, trade-off parameter α,β.**Initialization:** Freeze the final classifier layer $C_{t}=C_{s}$, and copy the parameters from $G_{s}$ to $G_{t}$ as initialization.**for**epoch=1**to**$T_{m}$**do**  Obtain self-supervised pseudo labels via Equation (4)  **for** iter=1 **to** $n_{b}$ **do**    # min-batch optimization    Sample a batch from target data and get the corresponding pseudo labels.    Update the parameters in $G_{t}$ via L in Equation (8).    Select label consistency samples and add them into memory bank.  **end for****end for**

## 4. Experiment

### 4.1. Datasets

In order to prove the effectiveness of LCCL, we conducted experiments on the following popular visual benchmarks.

VisDA-2017 [30] is a large simulation-to-real dataset, which is used for domain adaptation. There are more than 280,000 images in the field of training, verification and testing, covering 12 categories. The training images are generated from the same object in the simulation environment under different circumstances; the validation images are collected from MSCOCO. The experiment result is listed in Table 1.

Digits is a benchmark dataset for domain adaptation that focuses on digit recognition. It contains three domains, each of which consists of 10 categories. The three domains are: SVHN (S); MNIST (M); USPS (U). Following DANN [5], We use the training set of each domain to train our model, and report the recognition results on the standard test set of the target domain, shown as Table 2.

Office-31 [31] dataset is a common object in the office environment, such as keyboard, laptop and mouse. The dataset consists of three domains: Amazon, DSLR and webcam, each with 31 categories. The Amazon domain contains an average of 90 images per class, including 2817 images in total, which are taken by businesses in a clean background. The DSLR domain contains 498 low-noise high-resolution images (4288 × 2848), there are five objects in each category. The webcam domain includes 795 low resolution images (640 × 480) and it shows obvious noise, color and white balance artifacts.The experiment result is listed in Table 3.

### 4.2. Implementation Details

**Network architecture.** We ensure that the Source-only model used is the same as SHOT [9], which is the LeNet-5 [32] for digit recognition and resnet-50 [1] model for image classification pre-trained in the source domain. The model includes a feature extractor, a task-oriented classifier and a bottleneck layer between them. It should be noted that the feature dimension of the extracted picture after the bottleneck layer is 256. The BN layer is placed after the FC in the bottleneck layer and a weight normalization layer is used in the last FC layer.

### 4.3. Baselines

We compared LCCL with three types of baseline methods: (1) Source-only: ResNet [1]; (2) Unsupervised domain adaptation: DANN [5], MCD [16], ADR [38], CyCADA [39], rRevGrad + CAT [40], CDAN [33], TPN [17], SAFN [34], SWD [35], MDD [41], CAN [18], MCC [36]; (3) Source-free unsupervised domain adaptation: SHOT [9], PrDA [37], MA [20] and BAIT [21].

**Network hyper-parameters**. We implement our method under the PyTorch framework [42]. The source-only model, consistent with SHOT, which is a model trained with label smoothing technology. We train the whole network through back propagation, and the learning rate of the network is fixed at 1 × 10${}^{-3}$. Specifically, we use minibatch SGD with momentum of 0.9 and weight decay of 1 × 10${}^{-3}$. We set the learning rates of $\eta_{0}=1\;\times \;10^{-3}$ and $\eta_{0}=1\;\times \;10^{-2}$ for the visda-2017 dataset and other datasets, respectively. We further use the same learning rate scheduler $\eta =\eta_{0}\cdot {\left(1+10\cdot p\right)}^{-0.75}$ as SHOT to change the learning rate of the network. In addition, for all tasks, we set batch size to 64, α=0.3,β=0.5.

### 4.4. Overall Results

**Figure 3:**
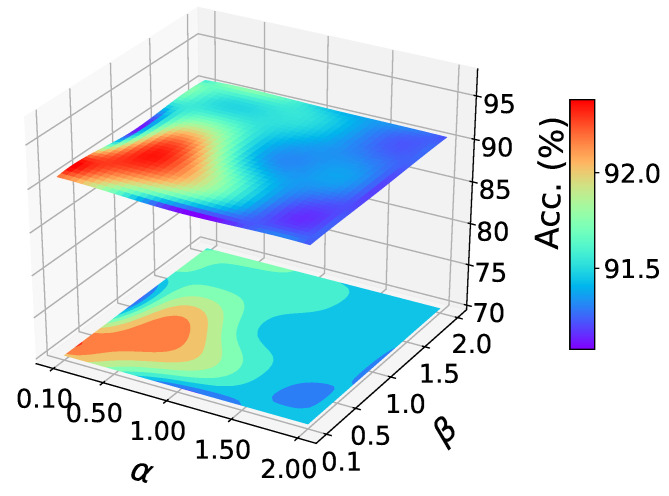
Parameter sensitivity study of task A→W, office-31.

**Results**. For data recognition, as shown in the Figure 2, LCCL obtains the best average accuracy for each task; however, the advantages are not obvious, mainly because the digital data set is relatively simple. For image recognition, as shown in the Figure 1 and Figure 3, we have achieved the highest average accuracy on office-31 and visda-2017 datasets, exceeding shot 0.6% and 0.5%, respectively. Specifically, we exceeded all other results on the four tasks in visda-2017. These convincing results show that our method has high performance, thanks to the use of pseudo label technology for self-training, and on the other hand, the use of contrastive learning has played an excellent performance on large datasets.

### 4.5. Experimental Analysis

**Ablation experiment**. In order to explore the impact of parts $L_{im}$, $L_{pl}$ and $L_{lccl}$ on our method, we conducted experiments on task office-31 dataset. It can be seen from the Table 4 that the model of source-only performs poorly. After adding $L_{im}$, the classification accuracy is greatly improved. With the loss of $L_{pl}$, the method can also achieve good results. The contrastive learning module further promotes the improvement of network performance.

**Parameter sensitivity analysis**. As shown in the figure, we studied the sensitivity of our method to parameters α and β. We randomly conducted experiments on A→W of office-31, and reported the results in the Figure 3. There are similar results on other tasks. It can be seen that the classification accuracy varies little in a large parameter range, which shows the stability of our method.

**Figure 4:**
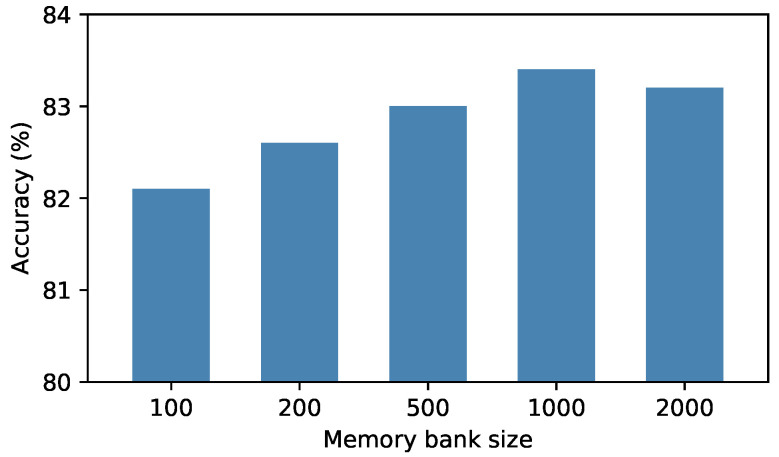
The impact of the memory bank size on visda-2017.

**Effect of memory bank size**. We conducted experiments on the VISDA dataset to explore the impact of memory bank size on adaptation, and reported the results in the Figure 4. It can be observed from the figure that the number of each class in the memory bank performs best at 1000, and the size is too low or too high is not particularly good. On the one hand, the memory bank capacity is too small and the number of samples saved is limited, so it is difficult to estimate the distribution of the whole sample well. On the other hand, if the capacity of the memory bank is too large, some redundant and outdated features will be added to the memory bank, making the result less satisfactory.

**Beyond SFUAD**. Our method can be used not only in source-free unsupervised domain adaptation, but also in traditional unsupervised domain adaptation. We add our method to the method DANN [5], and report the experimental accuracy in the Table 5. It can be seen that our method can significantly benefit DANN, which shows that our method is universal and has a wide application prospect.

**Figure 5:**
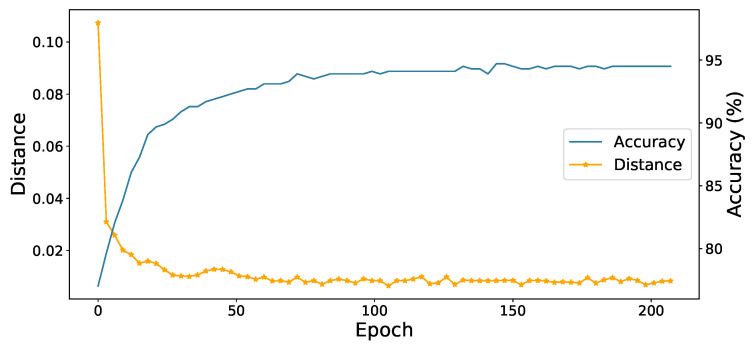
The accuracy of the model and average distance between sample and centroid with the epoch of training time on the A→D task in office-31.

**Convergence analysis**. In order to explore the convergence speed of network training and the influence of contrast learning on class aggregation. We show that the accuracy of the model and average distance between sample and centroid with the epoch of training time on the A→D task in office-31. From the Figure 5, we can see that the accuracy of the model increases steadily with the accumulation of training time, which shows that our method can select confident pseudo labels to promote network learning. At the same time, the distance within our class also decreases, which shows that our contrastive learning module can promote the same features to gather together in the feature space.

**Feature visualization**. In order to more clearly show that our method can adapt to the target domain very well, we further use t-sne [44] technology to visualize the classification effect of source only model and our final model. It is not difficult to see that in the source only model, different classes may mix up due to the offset between fields. Our method can better realize all kinds of separation, thanks to our contrastive learning, which can pull the samples of different classes far away and the samples of the same class closer.

**Figure 6:**
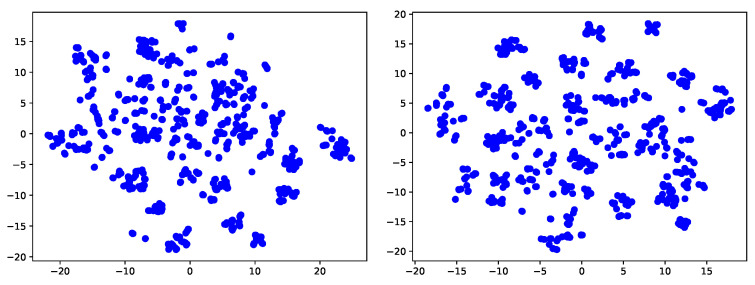
Feature visualization of task A→W, office-31. (**left**: source-only model; **right**: ours).

**Impact of label consistency (LC)**. In order to explore the impact of label consistency on the contrastive learning module, we added all features to the memory bank during the training process, and the results are shown in the Figure 6 and Table 6. By analyzing the data in the table, we can find that if there is no label consistent constraint, the experimental results will become worse, which shows that adding noise data to contrastive learning will damage the performance of our network.

## 5. Conclusions

In this paper, we propose a simple yet effective framework LCCL to address a practical setting called source-free unsupervised domain adaptation. LCCL merely needs the well-trained source model and offers the feasibility of unsupervised DA without access to the source data, which may be private issues. Specifically, LCCL learns the target-specific model by exploiting the information maximization and pseudo labeling, and introduces a memory bank that stores reliable samples with consistent labels for encouraging learn discriminative features at the class level. Extensive experiments on multiple tasks verify that LCCL achieves competitive and even state-of-the-art performance.

Future plan will address the limitations of the present work. The main limitation is that the proposed method is based on the contrastive learning. Due to the lack of source domain data and target domain labels, the proposed label consistency contrastive learning learns the distinguishability relationship with the historical model samples from the unmarked target samples. As a result, a memory bank is used to store historical sample feature, which increases the burden of memory to a certain extent. In the future, new source domain data and target domain labels will be collected using some specially designed experiments.

## Figures and Tables

**Table 1:** Classification accuracies (%) on VisDA-2017 dataset (ResNet-101).

Method	Plane	Bcycl	Bus	Car	Horse	Knife	Mcycl	Person	Plant	Sktbrd	Train	Truck	Per-Class
Source only	55.1	53.3	61.9	59.1	80.6	17.9	79.7	31.2	81.0	26.5	73.5	8.5	52.4
DANN [5]	81.9	77.7	82.8	44.3	81.2	29.5	65.1	28.6	51.9	54.6	82.8	7.8	57.4
CDAN [33]	85.2	66.9	83.0	50.8	84.2	74.9	88.1	74.5	83.4	76.0	81.9	38.0	73.9
SAFN [34]	93.6	61.3	84.1	70.6	94.1	79.0	91.8	79.6	89.9	55.6	89.0	24.4	76.1
SWD [35]	90.8	82.5	81.7	70.5	91.7	69.5	86.3	77.5	87.4	63.6	85.6	29.2	76.4
TPN [17]	93.7	85.1	69.2	81.6	93.5	61.9	89.3	81.4	93.5	81.6	84.5	49.9	80.4
MCC [36]	88.7	80.3	80.5	71.5	90.1	93.2	85.0	71.6	89.4	73.8	85.0	36.9	78.8
PrDA [37]	86.9	81.7	**84.6**	63.9	**93.1**	91.4	86.6	71.9	84.5	58.2	74.5	42.7	76.7
SHOT [9]	94.3	**88.5**	80.1	57.3	93.1	94.9	80.7	80.3	**91.5**	89.1	86.3	58.2	82.9
MA [20]	**94.8**	73.4	68.8	**74.8**	**93.1**	**95.4**	88.6	**84.7**	89.1	84.7	83.5	48.1	81.6
BAIT [21]	93.7	83.2	84.5	65.0	92.9	**95.4**	**88.1**	80.8	90.0	89.0	84.0	45.3	82.7
**LCCL**	92.8	86	78.7	60.4	92.9	93.9	87.0	81.1	**91.5**	**91.3**	**86.5**	**59.3**	**83.4**

**Table 2:** Classification accuracies (%) on **Digits** dataset (LeNet-5). S: SVHN, M:MNIST, U: USPS.

Method	S→M	U→M	M→U	Avg.
Source-only	70.2	88.0	79.7	79.3
ADDA [43]	76.0	90.1	89.4	85.2
ADR [38]	95.0	93.1	93.2	93.8
CDAN + E [33]	89.2	98.0	95.6	94.3
CyCADA [39]	90.4	96.5	95.6	94.2
rRevGrad + CAT [40]	98.8	96.0	94.0	96.3
SWD [35]	98.9	97.1	98.1	98.0
SHOT [9]	98.9	98.0	97.9	98.3
MA [20]	**99.4**	**99.3**	97.3	98.6
**LCCL**	**99.4**	98.8	**97.9**	**98.7**

**Table 3:** Accuracies (%) on Office-31 dataset (ResNet-50).

Method	A→D	A→W	D→W	W→D	D→A	W→A	Avg.
Source-only	68.9	68.4	96.9	68.2	99.1	67.4	76.1
DANN [5]	79.7	82.0	96.9	99.1	67.4	68.2	82.2
MCD [16]	92.2	88.6	98.5	100.0	69.5	69.7	86.5
CDAN [33]	92.9	94.1	98.6	100.0	71.0	69.3	87.7
MDD [41]	90.4	90.4	98.7	99.9	75.0	73.7	88.0
CAN [18]	95.0	94.5	99.1	99.6	70.3	66.4	90.6
MCC [36]	95.6	95.4	98.6	100.0	72.6	73.9	89.4
SHOT [9]	93.1	90.9	98.8	99.9	74.5	74.8	88.7
PrDA [37]	92.2	91.1	98.2	99.5	71.0	71.2	87.2
MA [20]	92.7	93.7	98.5	99.8	**75.3**	**77.8**	**89.6**
BAIT [21]	92.0	**94.6**	98.1	**100.0**	74.6	75.2	89.1
**LCCL**	**94.5**	92.2	**98.9**	99.9	**75.3**	75.1	89.3

**Table 4:** Ablation study of our method.

Datasets	Digits	Office-31	VisDA-2017
Source-only	79.3	76.1	52.4
+$L_{im}$	95.2	87.3	80.4
+$L_{im}$ + $L_{pl}$	98.3	88.6	82.9
+$L_{im}$ + $L_{pl}$ + $L_{lccl}$	98.7	89.3	83.4

**Table 5:** The benefits of our approach to DANN.

Method	A→D	A→W	D→W	W→D	D→A	W→A	Avg.
DANN [5]	79.7	82.0	96.9	99.1	67.4	68.2	82.2
DANN + $L_{lccl}$	**90.8**	**90.3**	**98.5**	**99.9**	**72.7**	**70.7**	**87.2**

**Table 6:** Impact of not label consistency. Accuracies (%) on office-31 dataset (ResNet-50).

Method	A→D	A→W	D→W	W→D	D→A	W→A	Avg.
Source only model	92.4	91.3	96.5	**99.9**	74.8	73.9	88.1
Ours	**94.5**	**92.2**	**98.9**	**99.9**	**75.3**	**75.1**	**89.3**

## Data Availability

Not applicable.
